# Simultaneous Determination of Matrine and Tinidazole in Compound Lotion by RH-HPLC Method

**DOI:** 10.1155/2013/185706

**Published:** 2013-07-18

**Authors:** Zhikui Yin, Suying Ma, Jincai Wang, Xiaojun Shang

**Affiliations:** ^1^School of Pharmacy, Xinxiang Medical University, Jin Sui Road, Xinxiang 453003, China; ^2^Department of Pharmacy, The First Affiliated Hospital of Xinxiang Medical University, Jian Kang Road, Weihui 453100, China

## Abstract

A simple, sensitive, and accurate RP-HPLC coupled with UV detector method was developed and validated for simultaneous determination of matrine and tinidazole in compound lotion. The chromatographic separation of the two compounds was carried out with a SinoChoom ODS-BP C_18_ column (5 **μ**m, 4.6 mm × 200 mm) analytical column, using a mobile phase consisting of 0.025 mol/L potassium dihydrogen phosphate (containing triethylamine 0.05%, v/v) and acetonitrile (80 : 20, v/v) at a flow rate of 1.0 mL/min. The detection was monitored at 210 and 310 nm for matrine and tinidazole, respectively. Total run time was 12 min, and the column was maintained at 25°C. The excipients in the compound lotion did not interfere with the drug peaks. The calibration curves of matrine and tinidazole were fairly linear over the concentration ranges of 10.0–100.0 **μ**g/mL (*r* = 0.9954) and 20.0–200.0 **μ**g/mL (*r* = 0.9968), respectively. The RSD of both the intraday and interday variations was below 1.5% for matrine and tinidazole. The proposed HPLC method was validated according to International Conference on Harmonisation and proved to be suitable for the simultaneous determination of matrine and tinidazole in compound lotion.

## 1. Introduction

Vaginosis is an inflammation of the vagina. It can result in discharge, itching, and pain and is often associated with an irritation or infection of the vulva. The infection is usually caused by *Candida albicans*, *Gardnerella, Trichomonas vaginalis, chlamydia, *and *Mycoplasma.* It is estimated that bacterial vaginosis is associated with a twofold increased risk of preterm birth and a sixfold increased risk of miscarriage [1]. Bacterial vaginosis is also found to be associated with an increased susceptibility to sexually transmitted infections, herpes simplex viruses, human papillomavirus, and human immunodeficiency virus [2, 3]. It is necessary to prepare an effective preparation for prevention and treatment of vaginosis.

**Figure 1:**
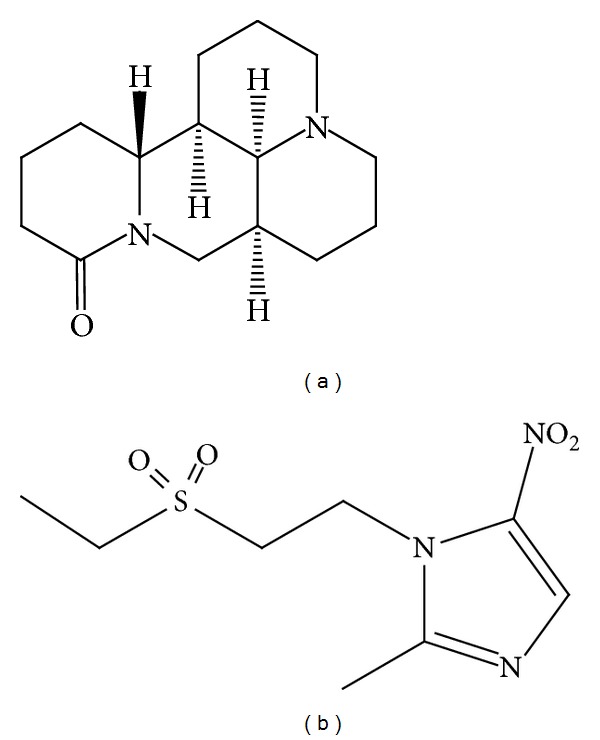
Chemical structures of matrine (a) and tinidazole (b).

*Sophora flavescens* Ait is a leguminous plant widely distributed in the northwestern region of China. It is an important Chinese herbal remedy that has been used for thousands of years in history. The important ingredient extracted from the herbal remedy was matrine whose chemical name is (7a*S*, 13a*R*, 13b*R*, 13c*S*)-dodecahydro-1*H*, 5*H*, 10*H*-dipyrido [2, 1 − *f* : 3′, 2′, 1′ − *ij*] [1,6] naphthyridin-10-one (Figure 1) [4, 5]. It has been officially listed in the Chinese Pharmacopoeia. Previous studies have indicated that matrine has a variety of pharmacological effects, including anti-inflammatory, anticancer, and action as a kappa opioid receptor and *μ*-receptor agonist [6–9]. It was often used to treatment of inflammatory disease and cancer with no obvious toxicity or side effect in clinical.

Tinidazole, chemically 1-(2-ethylsulfonylethyl)-2-methyl-5-nitro-imidazole, is a second-generation 5-nitroimidazole product (Figure 1). It is widely used for treatment and prevention of many protozoan infections caused by amoeba, giardia, and trichomonas because of higher efficacy and fewer adverse effects.

The compound lotion of matrine and tinidazole can play synergy roles in the effective prevention and treatment of vaginosis diseases.

HPLC methods have been widely used to determine matrine and tinidazole in samples at present [10–19]. To our knowledge, few HPLC methods have been developed in the literature for determination of matrine and tinidazole in lotion simultaneously. The literature was mainly focused on determination of the content of matrine and tinidazole one by one.

The aim of this study is to develop and validate a simple, rapid, sensitive, and reproducible HPLC method according to International Conference on Harmonisation to determine matrine and tinidazole simultaneously when combined in lotion with the advantages of shorter retention time and run time [20].

## 2. Experiment

### 2.1. Reagents and Chemicals

Tinidazole and matrine were received as gifts from Zhengzhou Yonghe Pharmaceutical Co., Ltd. (Zhengzhou, China). The two standards were of over 99.5% purity. HPLC grade acetonitrile and other analytical grade chemicals were purchased from Xinshiji Chemicals Co., Ltd. (Xinxiang, China). The deionized water in the study was purified with Smart2 Pure 12 UV/UF purification system (Thermo Fisher Scientific, USA).

### 2.2. Preparation of Matrine/Tinidazole Compound Lotion

Matrine/tinidazole compound lotion was prepared by dissolution method. Tinidazole (2 g) and matrine (1 g) were accurately weighted into a 100 mL glass beaker and then by dissolved with 40 mL ethanol. 20 mL glycerin and 0.5 g menthol dissolved with 10 mL ethanol were mixed thoroughly in tinidazole/matrine ethanol solution. After mixing, the solution was made up to 1000 mL with water in ultrasonic bath for 10 min, filtered, packed, and stored in the refrigerator at 4°C for further use. 

### 2.3. Liquid Chromatographic Conditions

The liquid chromatographic analyses were performed using a Shimadzu system that is comprised of an LC-20AT pump, SPD 20A UV-visible absorbance detector connected to Shimadzu Spin Chrome software. Chromatographic separation was achieved on a reversed-phase ODS-BP C_18_ column (5 *μ*m, 4.6 mm × 200 mm). An injection volume of 20 *μ*L was optimized in method via a Rheodyne syringe.

The mobile phase under isocratic mode was a mixture of acetonitrile-triethylamine (0.05%) in 0.025 mol/L potassium dihydrogen phosphate (20 : 80, v/v). The mobile phase was degassed by an ultrasonic bath and filtered through a 0.45 *μ*m membrane filter under vacuum. The eluents were detected at 210 nm from 0 min to 7.0 min and at 310 nm from 7.0 min to 12.0 min. The flow rate was 1.0 mL/min. All determinations were performed at ambient temperature 25°C. 

### 2.4. Preparation of Standard Stock Solutions and Working Solutions

Stock standard solutions were prepared with mobile phase separately to give a final concentration of 1.0 mg/mL for tinidazole and matrine. The combined standard solutions were prepared with the previous two solutions. Intermediate and working solutions were prepared by diluting stock solutions with the mobile phase. Calibration standard solutions were prepared in the concentration range of 10.0 to 100.0 *μ*g/mL for matrine and 20.0 to 200.0 *μ*g/mL for tinidazole and injected into the system in triplicate. The chromatogram peak area of each drug concentration was calculated. The regression of the drug concentration versus the peak area was obtained.

### 2.5. Quantification of Tinidazole and Matrine in Compound Lotion

One mL compound lotion was accurately transferred into a 10 mL volumetric flask and made up to volume with mobile phase. The solution was diluted appropriately to yield concentrations of matrine (50.0 *μ*g/mL) and tinidazole (100.0 *μ*g/mL). A 20 *μ*L aliquot of the sample solution was injected into the chromatographic system three times under optimized chromatographic conditions. The peak area was measured at 210 nm from 0 min to 7.0 min and at 310 nm from 7.0 min to 12.0 min for matrine and tinidazole, respectively. Drug concentrations of the samples were determined by interpolation from calibration plots of each drug previously obtained. 

### 2.6. Method Validation

The method was validated in terms of parameters of specificity, linearity, sensitivity, accuracy, precision, and reproducibility according to International Conference on Harmonisation.

The specificity of the method was demonstrated by comparing chromatograms of combined working solution (matrine 50.0 *μ*g/mL and tinidazole 100.0 *μ*g/mL), blank excipients sample without matrine and tinidazole, and equal concentrations samples of compound lotion made with the previous procedure. All the samples were analyzed and recorded to ensure the absence of interfering peaks.

The linearity of the method was evaluated with matrine and tinidazole working solutions at eight different concentrations. The concentration was 10.0–100.0 *μ*g/mL for matrine and 20.0–200.0 *μ*g/mL for tinidazole, respectively. All the samples prepared for linearity were injected into chromatographic system (*n* = 3). The responses were measured as peak area.

The sensitivity of the method was tested with limit of detection (LOD) and limit of quantification (LOQ). The LOD and LOQ were expressed as the analyte concentration which generates a signal corresponding to three and ten standard deviations, respectively, above the mean blank signal. 

The accuracy of the method was assessed by comparing the percent analyte recovered by the proposed method at three concentration levels (matrine 40.0, 50.0, and 60.0 *μ*g/mL and tinidazole 80.0, 100.0, and 120.0 *μ*g/mL).

The precision of the method was checked by repeatability of injection, repeatability (intraday), intermediate precision (interday), and reproducibility. Injection repeatability was studied by calculating percent relative standard deviation (% RSD) for ten determinations each of peak area of matrine (50.0 *μ*g/mL) and tinidazole (100.0 *μ*g/mL) performed on the same day. The same solutions were injected in triplicate for both intraday and interday variations.

## 3. Results

### 3.1. Method Validation

**Figure 2:**
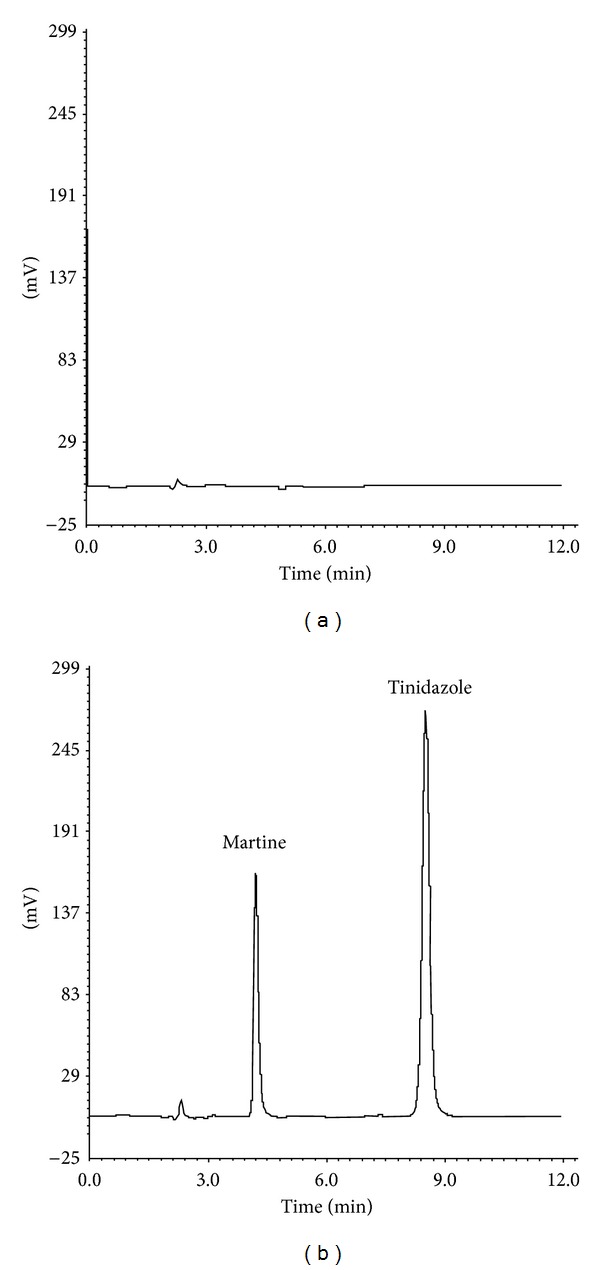
Typical HPLC chromatograms of (a) blank excipients sample and (b) compound lotion sample with matrine (50.0 *μ*g/mL) and tinidazole (100.0 *μ*g/mL).

The specificity was evaluated by analyzing blank excipients sample, combined working solution (matrine 50.0 *μ*g/mL, and tinidazole 100.0 *μ*g/mL), and equal concentrations samples of compound lotion. From the UV-visible spectra, matrine had maximum absorption at 210 nm and tinidazole had maximum absorption at 310 nm. Thus, 210 nm and 310 nm were selected as detection wavelengths. The typical HPLC chromatograms under optimum conditions were shown in Figure 2. The retention times of matrine and tinidazole at a flow rate of 1.0 mL/min were 4.90 min and 8.60 min, respectively. Analyte peaks were well resolved and free from tailing (<1.5 for both analytes). The excipients in the compound lotion did not interfere with the detection of matrine and tinidazole.

The calibration curves obtained by plotting peak area against concentration were linear over the concentration range of 10.0–100.0 *μ*g/mL for matrine and 20.0–200.0 *μ*g/mL for tinidazole at eight different concentrations, respectively. The correlation coefficient values were over 0.9950. Typical regression equations were calculated as follows: *y* = 28.27*x* + 25.08 for matrine and *y* = 33.99*x* + 134.7 for tinidazole, where *y* is peak area based on three parallel measurements and *x* is the concentration (*μ*g/mL) of matrine or tinidazole standard solution. The correlation coefficients indicate a good linear relationship between peak area and concentration over a wide range. 

Under the developed HPLC conditions, the LOD for matrine and tinidazole was 0.5 and 0.2 *μ*g/mL, respectively, which is the concentration that yields an S/N of 3, while LOQ was 1.0 and 0.5 *μ*g/mL, respectively. 

Mean recovery for matrine and tinidazole was 100.18 ± 0.65% and 99.92 ± 0.88%, respectively. The intra- and interday RSD values were lower than 1.5%. The low values of RSD revealed satisfactory precision and accuracy of this present method. Reproducibility was checked by having the samples analyzed by another analyst using the same instrument and the same laboratory. There was no significant difference between the RSD values indicating that the proposed method was reproducible. 

### 3.2. Content of Matrine and Tinidazole in Compound Lotion

Matrine and tinidazole were simultaneously determined with the proposed method in compound lotion. The results of the assay yielded 100.13 ± 0.53% for matrine and 99.86 ± 0.47% for tinidazole indicating that the method was selective and accurate for the simultaneous determination of matrine and tinidazole without interference from the excipients in the compound lotion dosage form. 

## 4. Discussion

This study was essentially focused on the simultaneous determination of coformulated matrine and tinidazole in compound lotion. Glycerol in the formulation can form film that could protect the skin. Menthol is not only used to reduce the pain stimulus, but it also promotes the penetration of the drug. Further study will be published in another research paper later. 

Matrine and tinidazole were soluble in organic solvents including methanol and acetonitrile. The use of mobile phase as extraction reagent provided minimal impurities and better separation.

Several mobile phase systems including methanol, acetonitrile, and buffer solutions of different proportions have been tested in this study. A mixture of acetonitrile-triethylamine (0.05%) in 0.025 mol/L potassium dihydrogen phosphate (20 : 80, v/v) under the isocratic elution system with a flow rate of 1.0 mL/min was selected as the optimum mobile phase for baseline separation, symmetrical peak, and shorter retention time. A UV detector was set at 210 nm for matrine and 310 nm for tinidazole. Under these conditions, elution of analytes was completed in less than 12.0 min. Retention times of matrine and tinidazole were 4.90 min and 8.60 min, respectively. The chromatograms were evaluated on the basis of peak areas of the two analytes. 

The method was validated according to ICH guidelines with the parameters of specificity, linearity, sensitivity, accuracy, precision, and reproducibility. The resolved analyte peaks without tailing between matrine and tinidazole showed the efficiency of the method to identify and determine each analyte at the same time with no interference. The accuracy, sensitivity, precision, and reproducibility data show that the method is accurate within the desired ranges. 

## 5. Conclusion

A simple, rapid, selective, and sensitive HPLC method has been developed and validated for the simultaneous determination of matrine and tinidazole when coformulated in compound lotion. The method will be helpful for simultaneous determination of matrine and tinidazole in compound lotion and can be reliably used by almost every drug laboratory.
